# A Novel Mouse Model of Alzheimer's Disease with Chronic Estrogen Deficiency Leads to Glial Cell Activation and Hypertrophy

**DOI:** 10.4061/2011/251517

**Published:** 2011-09-28

**Authors:** Annik Prat, Maik Behrendt, Edwige Marcinkiewicz, Sebastien Boridy, Ram M. Sairam, Nabil G. Seidah, Dusica Maysinger

**Affiliations:** ^1^Laboratory of Biochemical Neuroendocrinology, Clinical Research Institute of Montreal, 110 Pine Avenue West, Montreal, QC, H2W 1R7, Canada; ^2^Department of Pharmacology and Therapeutics, McGill University, 3655 Promenade Sir-William-Osler, Room 1314, McIntyre Medical Sciences Building, Montreal, QC, H3G 1Y6, Canada; ^3^Molecular Endocrinology Laboratory, Clinical Research Institute of Montreal, QC, Canada; ^4^Département de Médecine, Université de Montréal, Montréal, QC, Canada; ^5^Department of Medicine, Division of Experimental Medicine, Montreal, QC, Canada; ^6^Department of Physiology, McGill University, Montreal, QC, Canada

## Abstract

The role of estrogens in Alzheimer's disease (AD) involving *β*-amyloid (A*β*) generation and plaque formation was mostly tested in ovariectomized mice with or without APP mutations. The aim of the present study was to explore the abnormalities of neural cells in a novel mouse model of AD with chronic estrogen deficiency. These chimeric mice exhibit a total FSH-R knockout (FORKO) and carry two transgenes, one expressing the *β*-amyloid precursor protein (APPsw, Swedish mutation) and the other expressing presenilin-1 lacking exon 9 (PS1Δ9). The most prominent changes in the cerebral cortex and hippocampus of these hypoestrogenic mice were marked hypertrophy of both cortical neurons and astrocytes and an increased number of activated microglia. There were no significant differences in the number of A*β* plaques although they appeared less compacted and larger than those in APPsw/PS1Δ9 control mice. Similar glia abnormalities were obtained in wild-type primary cortical neural cultures treated with letrozole, an aromatase inhibitor. The concordance of results from APPsw/PS1Δ9 mice with or without FSH-R deletion and those with letrozole treatment in vitro (with and without A*β* treatment) of primary cortical/hippocampal cultures suggests the usefulness of these models to explore molecular mechanisms involved in microglia and astrocyte activation in hypoestrogenic states in the central nervous system.

## 1. Introduction

In the brain, estradiol is formed in neurons and a subpopulation of astrocytes by aromatase-mediated conversion of precursor androgens [1]. Estrogen deficiency was reported to accelerate *β*-amyloid (A*β*) plaque formation in an Alzheimer's disease (AD) mouse model combining an aromatase deficiency and an APP23 transgene [2]. There was no significant difference between the estrogen levels in APP23 and wild-type mice independent of age (3, 6, and 12 months); however, the estrogen levels in the brains of APP23-aromatase knockout mice were significantly reduced compared to age-matched ovariectomized APP23 mice. Furthermore, microglial cultures prepared from the brains of these APP23 mice showed impaired A*β* clearance and/or degradation [2]. 

Another model of estrogen imbalance was provided by follicule-stimulating hormone receptor (FSHR) knockout (FORKO) mice [3]. Our earlier studies showed that homozygous females were infertile, whereas males exhibited reduced fertility [4]. Similarly, inactivating mutations in the *FSHR* gene in women cause absolute infertility and amenorrhea [5]. Young and aged FORKO mice exhibit several biochemical and morphological abnormalities in the CNS, including mislocalization of chaperones and mitogen activated kinases, and hypertrophy of astrocytes, especially in aged mice [6–8]. These mice also develop metabolic syndrome [9] and cardiovascular abnormalities, which are risk factors for AD and other neurodegenerative disorders [10–13]. Although several studies indicated the contribution of reduced estrogens in circulation to the AD pathology, the role of this peripheral estrogen pool is still disputed [14–17]. The role of the local (brain) deficit of neuroestrogens is considered to be a critical player leading to CNS impairment associated with mild cognitive impairment (MCI) and AD in women [18–20]. 

Different studies suggest estrogens as anti-inflammatory agents [21, 22] and powerful modulators of glial cells [23, 24]. Depending on their activation states, microglia exhibit both neuroprotective (weakly activated) [25–27] and destructive (hyperactivated) roles [28–31]. Their role in inflammatory processes may affect AD development [32, 33]. Indeed, epidemiological studies reported that the use of nonsteroidal anti-inflammatory drugs could reduce the risk of AD [22, 34, 35]. However, activated resident microglia in a hypoestrogenic environment were unable to effectively clear A*β* deposits and possibly contributed to A*β* oligomerization [2, 36]. Although the internalization of A*β* is not limited to microglia, as both astrocytes and neurons are capable of uptake, microglia are the most efficient at this process in addition to retaining the capacity to degrade A*β* [37].

Several mouse models overexpressing human amyloid precursor protein (APP) with and without mutations were generated [38]. These mice developed amyloid plaques in different numbers, usually not before 6–9 months of age. Mouse models expressing APPsw (Swedish mutation) did not develop extensive amyloid aggregation before 18 months of age [39, 40]. However, double transgenic mice APPsw/PS1Δ9 (presenillin-1 lacking exon 9) exhibited elevated A*β*-levels at 6 months of age and extensive extracellular A*β*-deposits in the cortex and hippocampus at 9–12 months [40].

The objective of this study was to examine the impact of chronic hormone imbalance due to *FSHR* gene inactivation in the above APPsw/PS1Δ9 mouse model on the development of cortical and hippocampal plaque pathology and glial cell morphology. Accordingly, *FSHR* knockout (FORKO) mice with targeted disruption of the *FSHR* gene [3] were bred with the APPsw/PS1Δ9 line. In APPsw/PS1Δ9 and FORKO-APPsw/PS1Δ9 mice, plaques appeared as early as 3 months of age. Although their number did not differ significantly from the APPsw/PS1Δ9 mice later in life, FORKO-APPsw/PS1Δ9 mice exhibited significantly larger and more diffuse plaques in cortical and hippocampal regions. Moreover, enhancement of glial cell recruitment, size, and activation was seen as early as 3 months of age, as revealed by immunohistochemical labeling for both astrocytes and microglia. We further show that treatment of wild-type hippocampal primary cultures with the aromatase inhibitor, letrozole, also leads to glial cell hypertrophy and activation, suggesting that early changes in microglia and astrocytes may contribute to the progression of AD under conditions of local estrogen deficiency.

## 2. Materials and Methods

### 2.1. Mouse Strains

**Figure 1:**
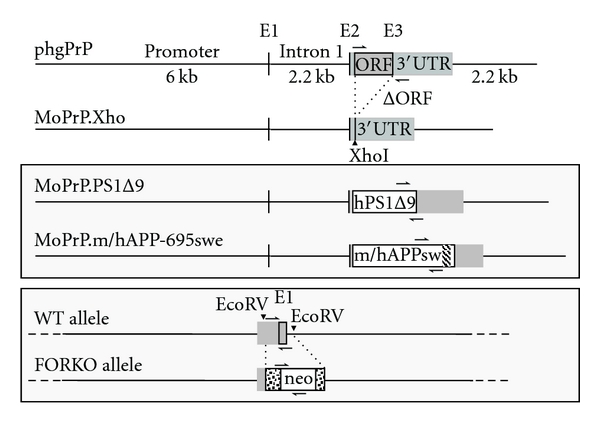
Generation of chimeric mice. A construct used to generate chimeric mice expressing human APP with Swedish mutation and hypoestrogenism due to the absence of FSH receptor. A murine prion protein (PrP) genomic fragment was modified to obtain the vector phgPrP [21]. A region containing 6 kb of promoter, exon 1 (noncoding), intron 1, and exon 2 (non-coding) was fused to the third and last exon (2008 bp) and 2.2 kb of downstream sequence. The subsequent removal of the PrP open reading frame (ORF) generated the vector MoPrP.Xho, in which the cDNA encoding PS1 lacking the exon 9 (amino acids 290–319) was subcloned [44]. Finally, the region encoding residues 592 to 622 from the mouse APP-695 cDNA was substituted by the corresponding human one (hatched area), which contains the Swedish KM to NL double mutation and the A*β*42 coding sequence. The resulting hybrid mouse/human cDNA was subcloned in MoPrP.Xho [11]. Half arrows indicate the position of the primers used for genotyping the mice. For complete inactivation of the FSH receptor gene, a 638 bp fragment containing part of the 5′ untranslated region, the coding region of exon 1 (101 bp), and about 129 bp of intron 1 was substituted by a 1.7 kb neomycin expression cassette (dotted box) [19]. A multiplex PCR using primers indicated by half arrows was used to genotype the offspring [16]. Black boxes: non-coding PrP exons 1 and 2; grey box: coding exon; ORF are boxed.

FORKO (*Fshr*−/−) males were produced by interbreeding 129T2/SV EmsJ *Fshr*+/− males and females [3, 4] and were then crossed with double transgenic females (C3HeJ x C57BL/6J F1 hybrid background). The two transgenes, APPsw and PS1Δ9, were previously described [39–41]. Both are expressed under the control of the mouse prion protein (PrP) promoter and are thus highly expressed in brain neurons and astrocytes. They were obtained by replacement of the PrP open reading frame and their structure is summarized in Figure 1 and its legend. F1 mice (*Fshr*+/−) were intercrossed. F2 *Fshr*+/+ mice were intercrossed, while *Fshr*−/− males were crossed with *Fshr*+/− females (*Fshr*−/− females are sterile). Only the male or the two females of each breeding were positive for the 2 transgenes. F3 mice positive for the two transgenes and either *Fshr*+/+ (1 out of 4) or *Fshr*−/− (1 out of 8) were analyzed. They were derived from 3 and 4 different breedings, respectively.

### 2.2. Genotyping

Genomic DNA from the tails of 3-week-old mice was extracted and tested by PCR amplification for the presence of the two transgenes, APPsw and PS1Δ9, and the genotype at the *Fshr* locus. The wild-type *PrP* gene and APPsw transgene were detected simultaneously using two specific sense primers, 5′-AACCTCATGGTGGTAGTTGG and 5′GATCTCTGAAGTGAATCTGGATG, respectively, and a common antisense primer, 5′-GCAAGAATGAGAACCACCTC. The combination of the 3 primers generated 645 and 420 bp products corresponding to the PrP wild-type allele (internal control) and APPsw transgene, respectively. Similarly, 3 primers were used to detect the wild-type *PrP* gene and the PS1D9 transgene. Two specific sense primers, 5′-AGCAACCAGAACAACTTCGT and 5′-GTTGCAGAGAATGATGATGG, and a common reverse primer, 5′-AGCAAAGAGCAACTGGTCTACT, generated 450 and 568 bp products for the PrP gene and PS1Δ9 transgene, respectively. Finally, wild-type *Fshr*+/+ alleles were detected using the sense 5′-AGTTCAATGGCGTTCCG and antisense 5′-CATGTCAGTAGTACATTAGAG primers (634 bp product) while the *Fshr*−/− alleles were detected using neomycin-specific primers, sense 5′-AAGGGACTGGCTGCTATTG and antisense 5′-AGAAAAGCGGCCATTTTC, to generate a 348 bp product.

### 2.3. Immunohistochemistry

Female mice were sacrificed at 3 or 6 months and brains were dissected. One half of each brain was frozen in isopentane at −35°C and kept at −80°C. A second half was fixed by immersion in 4% formaldehyde for 24 hrs, embedded in paraffin and sectioned into 5 *μ*m thick sections. Cryosections (10 *μ*m), obtained from the frozen half-brains, were fixed in 4% paraformaldehyde for 1 hr, washed in PBS, and incubated with rabbit antibodies directed against GFAP (AB5804; Chemicon International, Temecula, Calif, USA) or Iba1 (Wako Chemicals USA, Richmond, Va, USA) at 1 : 400 and 1 : 200 dilutions in 10% goat normal serum in PBS, respectively. GFAP labeling was revealed using the ImmunoCruz staining system (Santa Cruz, Calif, USA), while the Iba1 labeling was revealed by an Alexa 555 secondary antibody (A-21422; Molecular Probes, Invitrogen) used at a 1 : 100 dilution in 10% goat normal serum in PBS for 30 min at room temperature. For A*β* detection, the paraffin sections were deparaffinized by xylene, rehydrated by successive incubations in 100% to 50% ethanol solutions and PBS. Incubations were performed overnight at 4°C with rabbit antibodies, which are directed against the 8 first residues of A*β*40 (FCA18, a generous gift from F. Checler, Institute of Molecular and Cellular Pharmacology, Valbonne, France) or the C-terminal part of either A*β*1-40 (A8326 from Sigma, Mississauga, ON, Canada) or A*β*1-42 (66481G from BD Biosciences, San Jose, Calif, USA), all used at a 1 : 300 dilution in 10% goat normal serum in PBS. After 3 washes in PBS, the antibodies were revealed by using the Histomouse kit (Zymed Laboratories, San Francisco, CA, USA). To visualize senile plaques, sections were incubated for 1 hr in 1% aqueous thioflavin S (ThS; Sigma), dehydrated in 70% ethanol, cleared with Entellan new rapid mounting medium for microscopy (EM Science Harleco), and observed under UV light.

### 2.4. Image Analysis for Plaques and Glia Quantification

Immunostained sections were analyzed by means of an Olympus BX51 microscope, equipped with motorized stage and focus coupled by means of a CCD camera to a MCID Elite Image analysis system (Imaging Research Inc. St. Catherine, ON, Canada) according to the procedure described by Bell et al. [42, 43]. In summary, the total number of pixels in one field was taken as 1.0, and all immunofluorescent pixels from GFAP or Iba1 staining were taken as % of the total. Photomicrophotographs were collected from four to six sections per animal (*n* = 5–7 per experimental group). The use of preset shape was not desirable as plaques are not always symmetrical or uniform in size. To maintain consistency and determine plaque size, the outer border of the plaque was traced by the same individual. Plaque sizes were determined as perimeters or total surfaces, and mean values ± SD were calculated for all plaques within the same size range for the selected brain structures in both genotypes.

### 2.5. Primary Hippocampal Neural Cultures

Primary hippocampal neurons and glia from 5-day-old mouse pups (C57BL6; background of both the FORKO and APPsw/PS1∆9) were isolated, mechanically and enzymatically dissociated, counted and seeded (5 × 10^4^ cells/well) onto collagen-coated glass coverslips, and grown in a 24-well cell culture plate (Corning) at 37°C and 5% CO_2_ initially in phenol-free Dulbecco's Modified Eagle's Medium (Invitrogen) with 1 mM L-Glutamine (Sigma), sodium pyruvate, and penicillin-streptomycin-neomycin antibiotic cocktail (Invitrogen). On the second day in vitro, cells are cultured in Neurobasal A medium without phenol red (Invitrogen) supplemented with 2% (v/v) B-27 supplement (Invitrogen), 1% (v/v) penicillin-streptomycin-neomycin PSN (Invitrogen), and 1mM L-Glutamine (Sigma). Cells were treated on day 5 in vitro with letrozole (1 *μ*M) for 24 hrs.

### 2.6. 3-(4,5-Dimethylthiazol-2-yl)-2,5-Diphenyl Tetrazolium Bromide (MTT) Assay

After various time intervals, 50 ul of phosphate buffer saline (PBS; pH 7.2) containing MTT (5 mg/mL) was added to each well, resulting in a final MTT concentration of 0.5 mg/mL. Cells were incubated at 37°C with 5% CO_2_ and >95% relative humidity for 180 min, after which media was removed. DMSO was added to lyse the cells and dissolve the formazan produced. Triplicates from each well were collected into a 96-well plate (Sarstedt) and the absorbance at 595 nm of each well was measured using a Benchmark microplate reader (Bio-Rad, Mississauga, ON, Canada). Data was tabulated and graphed using Microsoft Excel 2007.

### 2.7. Immunocytochemistry

Following treatment, cells were fixed in 4% paraformaldehyde for 15 min at room temperature (RT), followed by three washes for 5 min each with phosphate buffer saline (PBS, pH 7.2); the samples were permeabilized with 0.1% triton-X-100 at RT for 5 min, followed again by three washes in PBS. Nonspecific binding sites were blocked for 60 min at RT with 10% goat serum (Sigma) diluted in PBS. The samples were then incubated overnight at 4°C with the primary antibodies (Rat anti-macII, 1 : 500 gift from collaborator; rabbit anti-GFAP, 1 : 250, DakoCytomation; mouse anti-*β*_III_-tubulin, 1 : 100, Chemicon). Following the incubation, samples were washed three times in PBS and incubated for 60 min at RT with the secondary antibodies (antirat Rhodamine red, 1 : 500, Jackson Laboratories; antirabbit AlexaFluor 594, 1 : 1000, Molecular Probes; antimouse AlexaFluor 594, 1 : 1000, Molecular Probes). Following three rinses in PBS, the samples were counterstained with Hoechst 33258 (Molecular Probes), rinsed once with distilled water, and mounted onto glass slides using Aqua-Poly/Mount (Polysciences). Fluorescence images were acquired at 63x with a Leica DFC350FX monochrome digital camera connected to a Leica DMI4000B inverted fluorescence microscope. Images were acquired and pseudocolored using Leica Application Suite (LAS) software.

### 2.8. Statistical Analysis

All data were expressed as mean SEM and analyzed by one-way ANOVA. When a significant effect was obtained with one-way ANOVA, student's *t*-test was used for analyzing the significance of the difference between two means. To test for interactions between genotype and gender for each behavioral paradigm, two-way factorial ANOVA was employed. A *P* value < 0.05 was considered to be statistically significant, unless stated otherwise.

## 3. Results

### 3.1. Generation of the Mouse Model FORKO-APPsw/PS1∆9

To analyze the impact of reduced estrogens on AD development, we generated mice carrying the APPsw and PS1∆9 transgenes in either a wild type or FORKO background (Figure 1). For this, double transgenic APPsw/PS1Δ9 females exhibiting increased production of both A*β*40/42 peptides [40] were crossed with FORKO males [3]. In all the mice analyzed, the transgenes were kept at the heterozygote level to avoid any phenotype related to their insertion site in the genome. In view of the sterility of FORKO females, the high incidence of lost litters, and the need to select for alleles of three loci, a large number of crossings were required over three generations, representing four years of work, to obtain sufficient mice for the present study (see section 2). The FORKO-APPsw/PS1∆9 mice did not show marked phenotypic differences from the parental lines, FORKO or APPsw/PS1∆9, up to at least 6 months of age.

### 3.2. Analysis of Amyloid Plaque Deposits in APPsw/PS1∆9 and FORKO-APPsw/PS1∆9 Mice

**Figure 2:**
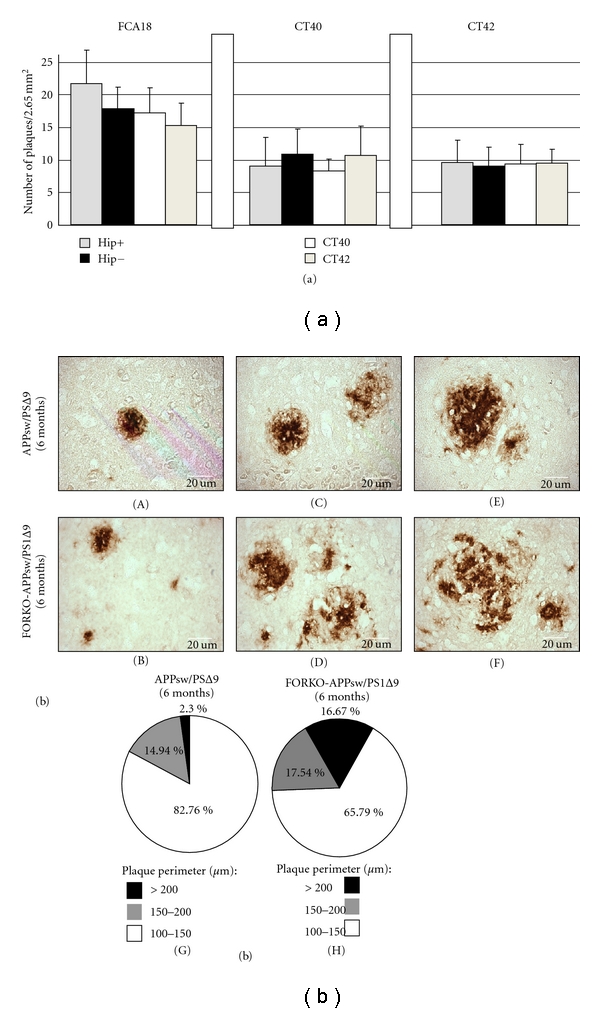
Six-month-old sex-hormone-imbalanced FORKO-APPsw/PS1∆9 mice show similar plaque localization and number but difference in plaque appearance and size compared to mice coexpressing APPsw/PS1∆9 alone. (a) Six-month-old sex-hormone-imbalanced FORKO-APPsw/PS1∆9 mice show no significant changes in plaques number compared to mice coexpressing APPsw/PS1∆9 alone. APPsw/PS1∆9 and FORKO-APPsw/PS1∆9 mice were examined by immunocytochemical staining for A*β* peptide with the polyclonal antibody anti-A*β*40/42 (FCA18), anti-A*β*40 (CT40), or anti-A*β*42 (CT42). Quantification of plaques number was achieved by plaque counting on sagittal brain sections of hippocampus and cortex in APPsw/PS1Δ9 (+/+; *n* = 6) and FORKO-APPsw/PS1∆9 (−/−; *n* = 6). (b) Presented is anti-A*β*40/42 (FCA18) immunostaining of sagittal brain sections from APPsw/PS1∆9 (*n* = 6; (A), (C), and (E)) and FORKO-APPsw/PS1Δ9 (*n* = 6; (B), (D), and (F)). By 6 months of age, both mice strains had developed A*β* plaques in typical areas of the brain, such as ((A) and (B)) the frontal cortex, ((C) and (D)) the parietal cortex, and ((E) and (F)) the hippocampus. Note that many more relatively large and diffuse plaques are found in chimeric mice compared to those in APPsw/PS1∆9 brains. Scale bar in all panels is 20 um. ((G) and (H)) Quantification of the plaques perimeter revealed 16.67 % of large, 17.54 % of medium, and 65.79 % of small plaques in FORKO-APPsw/PS1∆9 (H) compared to 2.3 % of large plaques, 14.94 % of medium and 82.76% of small plaques in APPsw/PS1∆9 (G).

Mouse models with varying APP mutations have not shown very extensive amyloid aggregation before 18 months of age [39, 40]. Studies of the double transgenic APPsw/PS1∆9 mice provided evidence for elevated A*β*-levels in 6-month-old mice, but extensive extracellular A*β*-deposits in the cortex and hippocampus were described in 9–12-month-old mice [40]. We focused on 6-month-old mice, as this period corresponds to an early stage in disease development. The nature of deposits in APPsw/PS1∆9 mice and the FORKO-APPsw/PS1∆9 mice was assessed by staining sagital sections with a rabbit polyclonal antibody FCA18, which recognizes both A*β*40 and A*β*42 [45], as well as with A*β*40 (CT40)- and A*β*42 (CT42)-specific antibodies (Figure 2(a)). There was no significant difference in the number of plaques revealed by the FCA18, CT40, or CT42 antibodies, as seen by the quantification in Figure 2(a). However, upon careful examination of the plaques using FCA18 antibodies we noted a marked difference in sizes and compactness (or density) of the immunopositive plaques (Figure 2(b) and Supplementary Figure 1 in Supplementary Material available online at doi: 10.4061/2011/251517). Both mouse strains have a large number of A*β*-aggregates in the frontal association cortex (Figure 2(b), (A) and (B)), as well as in the medial and lateral parietal sensory cortex (Figure 2(b), (C) and (D)) and the hippocampus (Figure 2(b), (E) and (F)). However, the perimeters of the plaques found in the brains of FORKO-APPsw/PS1∆9 are significantly larger and less dense, as revealed by the staining pattern, in comparison with APPsw/PS1∆9 transgenic mice, concurring with observations from Borchelt et al. [40]. Quantitative data regarding the image analyses of immunoreactive plaques are presented in pie charts and clearly show plaques in cortical regions with larger perimeters (17% of plaques >200 um) in FORKO-APPsw/PS1∆9 than in APPsw/PS1∆9 littermates (2% of plaques >200 um). These differences were not noticeable at 3 months of age given the relatively sparse deposition of plaques in both transgenic mouse strains (data not shown).

Aside from the brain regions in which plaque formation was commonly detected (i.e., cortex and hippocampus) in the APPsw/PS1∆9 and FORKO-APPsw/PS1∆9 mice, amyloid deposits develop in the olfactory bulb and occipital lobe (Supplementary Figure 2). Interestingly, even subcortical areas like the cerebellum, a brain region mainly considered to be devoid of A*β*-aggregates in most other animal models [46], had a low number of small size plaques (Supplementary Figure 2, (i)–(l)).

### 3.3. Astrocyte Hypertrophy and Microglial Activation

**Figure 3:**
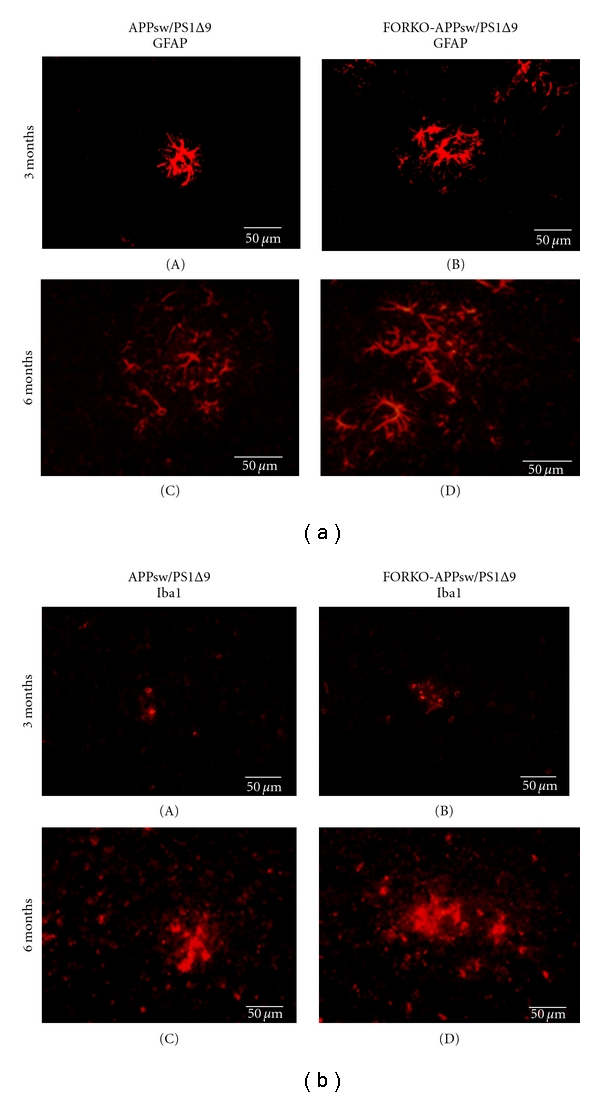
Sex-hormone-imbalanced 6-month-old FORKO-APPsw/PS1∆9 mice show enhanced number and hypertrophy of astrocytes and microglia in the vicinity of plaques in the hippocampus. Immunostaining for astrocytes and microglia was performed in representative areas of the cortex using polyclonal antibodies for GFAP (a) and MacII in (b) in APPsw/PS1∆9 mice and FORKO-APPsw/PS1∆9. Note that astroglia and microglia show an enlargement and increase in number, suggesting glial activation and inflammation in both APPsw/PS1∆9 FORKO-APPsw/PS1∆9 mice, more strikingly in the latter model. Scale bar measures 50 *μ*m.

Immunostaining with antibodies against GFAP and Iba1/Mac II was used to assess the morphology of astrocytes and microglia in FORKO-APPsw/PS1∆9 and in APPsw/PS1∆9. At 6 months of age, there were no marked differences between the two genotypes: (i) GFAP-positive astroglia were enlarged and abundant in FORKO-APPsw/PS1∆9 mice and in APPsw/PS1∆9 mice (Figure 3), and (ii) Iba1/MacII-positive microglia were also enlarged and exhibited morphologies reminiscent of highly activated states. Differences between the strains at 3 months of age were not apparent either (Figures 3(a), (A) and (B) and 3(b), (A) and (B)).

**Figure 4:**
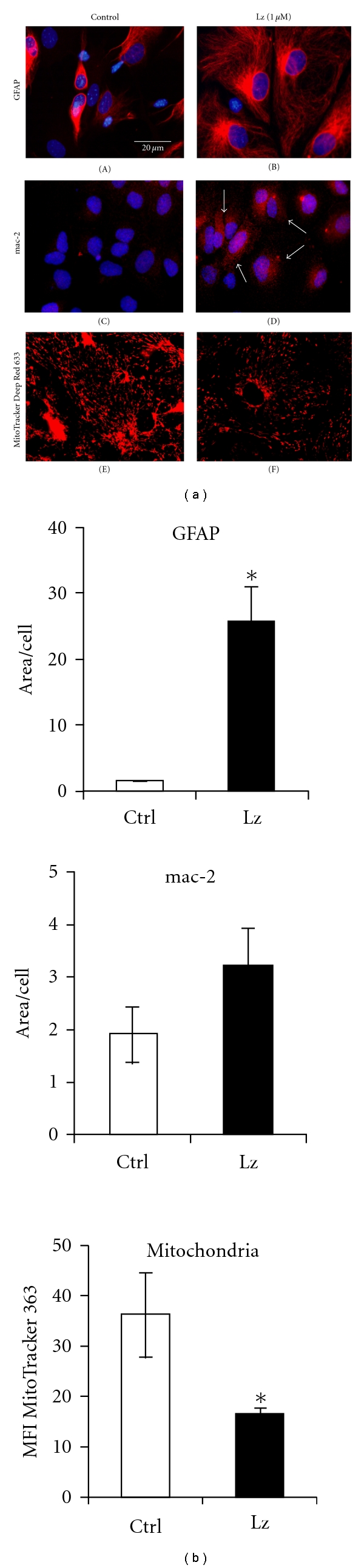
Immunocytochemical assessment of neuronal and glial status in primary hippocampal cultures treated with letrozole. Primary dissociated hippocampal cultures were treated with letrozole (1 *μ*M, 24 hrs), following which immunocytochemical analysis was performed for cell-specific markers. (a) Neurons ((A) and (B)) were stained with antibodies against beta III tubulin (1 : 100), astrocytes ((C) and (D)) with anti-GFAP (1 : 500), and microglia ((E) and (F)) with anti-Mac-2 (1 : 350). Note the marked astrocyte hypertrophy in letrozole-treated cells. Arrowheads indicate enlargement of the neuronal processes following Lz treatment. Arrows in (F) outline activated microglia, which exhibit increased mac-2 staining. ((G) and (H)) MitoTracker Deep Red 633 staining revealed truncated mitochondria in Lz-treated cells compared with control, indicative of increased mitochondrial fission and potentially mitochondrial dysfunction. Scale bar (20 *μ*m) in first panel is representative for all panels. (b) Quantification of stained area per cell for neurons (*β*_III_-tubulin), astrocytes (GFAP), and microglia (mac-2) following letrozole treatment.

Multiple attempts to set primary neural cortical and hippocampal cultures from FORKO-APPsw/PS1∆9 mice failed because the cells from these chimeric mice detached within 24–30 hours, whereas the same conditions were suitable for control cultures with similar genetic background (C57BL6). Interestingly, while performing brain dissection for primary cultures, the brains of chimeric mice appeared less structured and less solid suggesting that connections between neural cells were reduced in these brains. Therefore, we used primary hippocampal dispersed cultures from wild-type mice and exposed them to letrozole, an aromatase inhibitor, for 24 hrs to assess the changes in the neural cells with suppressed estrogen synthesis. Hippocampal neural cells treated with 10^−6^ M letrozole stably attached to the plastic surface, exhibiting similar viability to control cultures (Supplementary Figure 3). Morphological changes in glial state and neurons in this in vitro model are shown (Figures 4(a) and 4(b)). *B*_III_-tubulin-positive letrozole-treated neurons exhibited significant enlargement of their processes compared to the control (Figure 4(a), (A) and (B)), while highly activated microglia showed increased immunoreactivity for mac-2 following letrozole treatment (Figure 4(a), (E) and (F)) and GFAP-immunopositive astrocytes were significantly larger in letrozole treated cells compared with untreated controls (Figure 4(a), (C) and (D)), as confirmed by quantitative analysis (Figure 4(b)).

Given that metabolic dysfunction and oxidative stress are early events in AD [33, 47, 48] and that many of the neuroprotective effects of estrogens have been attributed to rescue of mitochondrial function [49] we also decided to investigate mitochondrial status following estrogen deprivation. In primary hippocampal neural cultures with and without letrozole treatment for 24 hrs, we labeled mitochondria using MitoTracker Deep Red 633 (Figure 4 G-H). Images captured with confocal microscopy reveal significant mitochondrial fragmentation and loss following letrozole treatment, which has been shown to correlate with a state of mitochondrial and synaptic dysfunction in the CNS [50].

## 4. Discussion

Estrogens in the CNS play both neuroprotective and neuromodulatory roles [51–53], which was associated with the increased incidence of age-related neurodegenerative diseases in women [54, 55]. The controversies over the concentrations of peripheral and brain sex hormones in cases with mild cognitive impairments (MCI) and AD are still not resolved [15, 17, 56–58]. Several clinical trials are ongoing and the results confirming or refuting the role of declining endogenous estradiol and effectiveness of hormone replacement therapy in early or even undiagnosed AD are eagerly awaited. 

Results from our studies present a new mouse model with three mutated loci (FORKO-APPsw/PS1Δ9), conferring sex hormone imbalance and AD pathology. The results clearly show that FORKO-APPsw/PS1∆9 mice have larger and more diffuse plaques than the double transgenic model, in addition to significant hypertrophy and activation of microglia and astrocytes, corroborating findings that estrogens are potent glia regulators [17, 22, 59, 60] whose state may be either beneficial or destructive in the CNS [61], particularly in aging. 

FORKO females exhibit hypergonadotropic-hypogonadism with high levels of circulating FSH and LH similar to the postmenopausal state in women [62]. In menopause, dramatic and rapidly occurring cessation of gonadal function leads to a loss of estrogens in women around 51 years of age [44, 63]. In contrast to menopause in women, men do not experience irreversible arrest of reproductive capacity in old age and aging in men is characterized by a gradual and slow decline in testosterone with half bioavailable testosterone levels at 75 years of age relative to younger men [64, 65]. Consequent to gender differences in senescence, significant differences are also found in the incidence, timing, and susceptibility of age-related conditions, such as osteoporosis, obesity, cardiovascular diseases, type 2 diabetes, depression, anxiety, insomnia, and memory impairment [44, 63, 64, 66–76]. 

Results from the triple mutant mouse model FORKO/APPsw/PS1∆9 presented here support the notion that the reduced circulating levels of estradiol do not significantly contribute to the plaque numbers but rather influence their compactness and size. Moreover, subphysiological concentrations of estrogens in this mouse model of Alzheimer's disease (FORKO/APPsw/PS1∆9) show significant reactivity of both astrocytes and microglia. 

Both glia and neurons can synthesize estrogens in human and rodent brains. The synthesis is catalyzed by the enzyme aromatase, widely distributed in the brain regions including those affected in AD, for example, basal forebrain, cerebral cortex, and hippocampus [1]. Although the positron emission tomography of human subjects has given us some insight into the general map of the human aromatase expression, the detailed distribution map still remains to be completed. Several studies point towards the significance of the local production of estradiol by aromatase conversion of testosterone in the brain, particularly on the neurons in the hippocampus [1, 16, 20, 77, 78]. Moreover, estrogens exert inhibitory effects on inflammation as well as, microglial and astroglial activation in several brain regions including cerebral cortex and hippocampus [22, 26, 59, 60, 79, 80]. Specifically, estrogen downregulates the release of inducible nitric oxide synthase (iNOS), tumour necrosis factor alpha (TNF*α*), interleukin-6 (IL-6), and interferon-gamma inducible protein-10 (IP-10) in cultured astrocytes activated with lipopolysaccharide (LPS) [81, 82]. Moreover, it was shown previously that long-term estrogen deprivation accelerates the appearance of highly reactive microglia at amyloid deposits, an event that was prevented by estradiol replacement [22]. 

The link between inflammation, microglia, and AD pathology was proposed long ago [34], but studies investigating the origin of microglia associated with plaques in AD are more recent and bring out different observations [83–85]. Microglia are attracted to the region surrounding senile plaques in human and rodent tissues [86–92] and A*β*40 and A*β*42 isoforms serve as chemoattractants. Conversely, chronic inflammation of the central nervous system associated with hyperactive states of glial cells is a risk factor for AD. We did not explore the origin of microglia but we show that they play a role in determining the size and shape of plaques in both APPsw/PS1∆9 mice and in the FORKO-APPsw/PS1∆9 mice. Importantly, this diffuse plaque state created by activated microglia unable to efficiently clear A*β* aggregates may lead to the release of soluble forms of A*β* from these plaques, which would therefore act as reservoirs, as suggested by others [93]. Recent studies by Selkoe suggest that amyloid fibrils from plaques are not as deleterious as soluble A*β*, particularly in its dimeric form [94, 95]. Inflammation in AD and specifically the effects on microglial activation in relation to A*β* removal are controversial: some claim it as a self-reinforcing positive feedback loop that potentiates the damaging effects of A*β*, while others claim that activated microglia are neuroprotective [96, 97]. 

To address the question of astrocyte hypertrophy and microglia activation in states of reduced estradiol synthesis by neural cells, we performed experiments with primary cultures. Dispersed primary hippocampal and cortical cultures from the brains of FORKO-APPsw/PS1Δ9 were not viable, therefore, for in vitro studies, we used wild-type mice and a common pharmacological approach to reduce estradiol synthesis using letrozole which inhibits aromatase enzymatic activity [16, 77]. Primary dissociated hippocampal cultures treated with letrozole show marked glial hypertrophy and microglial activation suggesting that such cultures could be useful for screening new therapies for neurological disorders accompanied by gliopathies in hypoestrogenic states. Our results show mac-2-positive staining in the hippocampus of chimeric mice, a marker of activated microglia, which is in accordance with the observation of mac-2 upregulation in wild-type cultures with pharmacologically inhibited aromatase by letrozole, previously used to study synaptic plasticity in hippocampal primary cultures [16, 77, 78, 98]. This experimental paradigm also allowed an assessment of mitochondrial morphological and functional status in neural cells, given that mitochondria respond to estradiol treatment [49].

Mitochondria are organelles known to be affected in AD [99]. It was reported that impairments in mitochondrial fission and fusion can lead to mitochondrial dysfunction and synaptic deficits in AD [100, 101]. Estradiol acts through mitochondria to exhibit neuroprotection. Our finding that estradiol deprivation leads to mitochondrial fragmentation and loss is not surprising, but it is among the first to show a correlation between glial activation and mitochondrial fragmentation in states of estrogen deprivation and sex-hormone imbalance. These observations suggest that estrogen may modulate mitochondria to regulate glial cell activation and hypertrophy. 

In summary, results from the present study support the notion that activated glia in a hypoestrogenic state could potentiate the effects of inflammation, in addition to enhancing A*β* release from plaques, and A*β* oligomerization. These forms of A*β* are major soluble neurotoxicants [102], therefore it is plausible that in the aged chimeric brains, cell loss could be due to enhanced oligomer formation. Results from the pilot studies indicate that the levels of A*β* in the brain of chimeric mice are not significantly different from APPsw/PS1∆9, but the kinetics of A*β* monomer and soluble oligomer formation remains to be determined. In order to detect and quantify small differences between these two genotypes with limited CSF and blood samples, we are currently developing nanobased sensors which could enable measurements of A*β* monomers and oligomers in small volumes of these biological samples.

In addition, results from our chimeric FORKO/APPsw/PS1∆9 mouse model are in line with both the Brinton hypothesis [103, 104] and the critical window hypothesis [105]. Although the two interrelated hypotheses, for example, (1) healthy cell bias of gonadal hormone action hypothesis [103, 104] and (2) the critical window hypothesis [105], reconcile previous controversies, they do not yet explain the positive versus negative effects of estrogens on cognition. Ongoing clinical studies with initiated estrogen therapy in young women, together with mechanistic investigations, will provide new insights to resolve the controversial issues. Our studies are also in line with elegant work by Garcia-Segura's group suggesting that the brain aromatase in the central nervous system and the local production of estradiol in the brain play a critical role in maintaining normal physiological central nervous system functions, including those in neurons and glial cells affected in AD [59, 79]. A particularly attractive feature of future studies is to explore the selective estrogen receptor modulators to normalize hyperactive glial cells and the release of proinflammatory cytokines and chemokines in inflammation, not only in AD, but also in other neurological disorders accompanied by excessive gliosis [80].

## Supplementary Material

Supplemental Figure 1: A*β* plaques are found in the cortex and hippocampus in 6-months old animals of both mouse strains. APPsw/PS1Δ9 (*n* = 6; A/B, E/F, I/J) and FORKO-APPsw/PS1Δ9 (*n* = 6; C/D, G/H, K/L) mice were examined by immunocytochemical staining for A*β*40 peptide with the polyclonal antibody FCA18. (A-D) Frontal cortex; (E-H) Parietal cortex and (I-L); Hippomcampus. (∗) points the plaques presented in lover and higher magnification, arrows indicate the corresponding picture in a higher magnification (Scale bar in A, C, E, G, I, K measures 100 *μ*m, in B, D, F, H, J, L 50 *μ*m).
Supplemental Figure 2: A*β* plaques occur in other regions aside from cortex and hippocampus in 6-months old animals of both mouse strains. APPsw/PS1Δ9 (*n* = 6; A/B, E/F, I/J) and FORKOAPPsw/PSΔ9 (*n* = 6; C/D, G/H, K/L) mice were examined by immunocytochemical staining for A*β*40 peptide with the polyclonal antibody FCA18. (A-D) Olfactory bulb, (EH) Occipital cortex and (I-L) Cerebellum. (∗) points the plaques presented in lover and higher magnification, arrows indicate the corresponding picture in a higher magnification (Scale bar in A, C, E, G, I, K measures 100  *μ*m, in B, D, F, H, J, L 50  *μ*m).
Supplemental Figure 3: Letrozole (10–6 M) is non-toxic to primary neural cultures. Primary neural cell viability, as measured using the MTT assay, was made relative to untreated control cells (=100%). Mean values and standard error of the means for triplicate measurements from three independent experiments (*N*=9) are shown. Ctrl=Control, Lz=Letrozole.
