# Supplementary figures and images for: A Novel Mouse Model of Alzheimer's Disease with Chronic Estrogen Deficiency Leads to Glial Cell Activation and Hypertrophy
Source: J Aging Res. 2011 Sep 28;2011:251517. doi: 10.4061/2011/251517 (PMC3182380; doi:10.4061/2011/251517)

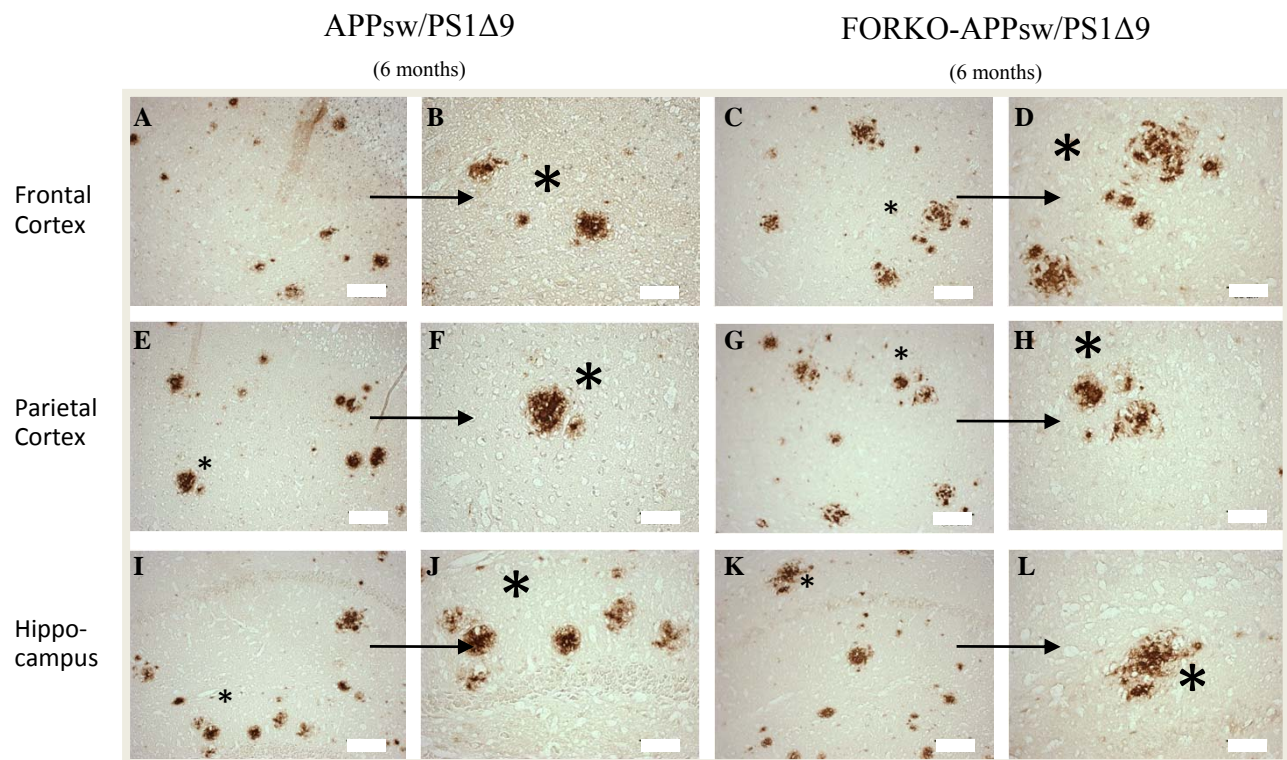

**Supplemental Figure 1**

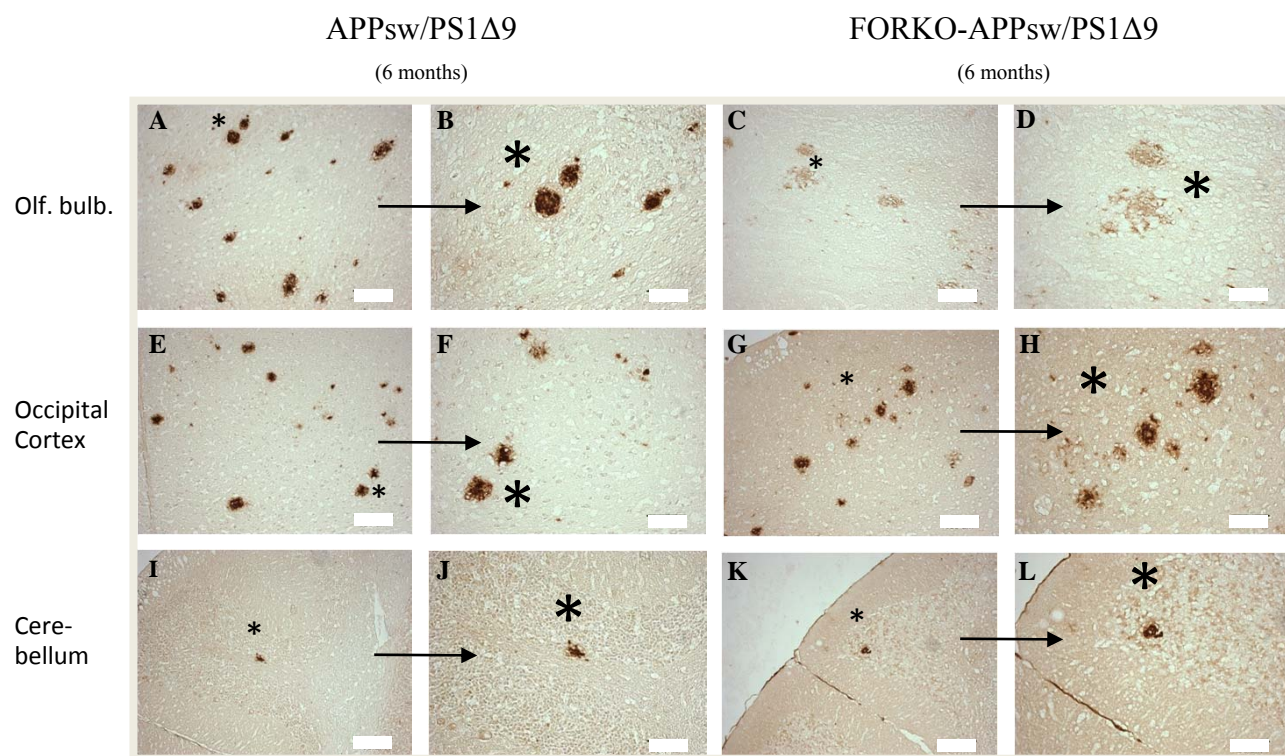

**Supplemental Figure 2**

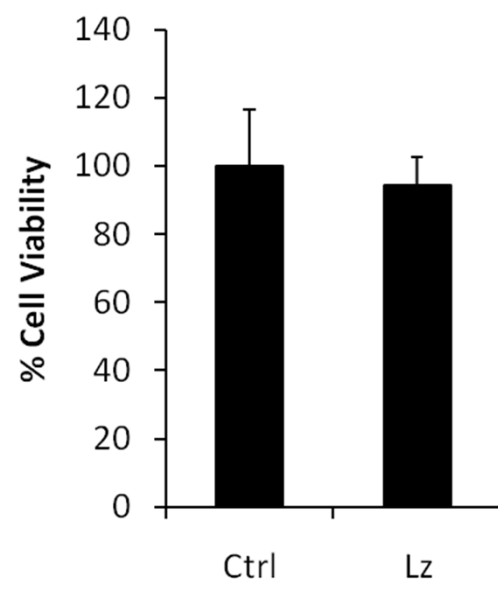

**Supplemental Figure 3**

Supplement: Supplementary file 1 — Supplemental Figure 1: Aβ plaques are found in the cortex and hippocampus in 6-months old animals of both mouse strains. APPsw/PS1Δ9 (n = 6; A/B, E/F, I/J) and FORKO-APPsw/PS1Δ9 (n = 6; C/D, G/H, K/L) mice were examined by immunocytochemical staining for Aβ40 peptide with the polyclonal antibody FCA18. (A-D) Frontal cortex; (E-H) Parietal cortex and (I-L); Hippomcampus. (∗) points the plaques presented in lover and higher magnification, arrows indicate the corresponding picture in a higher magnification (Scale bar in A, C, E, G, I, K measures 100 μm, in B, D, F, H, J, L 50 μm). Supplemental Figure 2: Aβ plaques occur in other regions aside from cortex and hippocampus in 6-months old animals of both mouse strains. APPsw/PS1Δ9 (n = 6; A/B, E/F, I/J) and FORKOAPPsw/PSΔ9 (n = 6; C/D, G/H, K/L) mice were examined by immunocytochemical staining for Aβ40 peptide with the polyclonal antibody FCA18. (A-D) Olfactory bulb, (EH) Occipital cortex and (I-L) Cerebellum. (∗) points the plaques presented in lover and higher magnification, arrows indicate the corresponding picture in a higher magnification (Scale bar in A, C, E, G, I, K measures 100 μm, in B, D, F, H, J, L 50 μm). Supplemental Figure 3: Letrozole (10–6 M) is non-toxic to primary neural cultures. Primary neural cell viability, as measured using the MTT assay, was made relative to untreated control cells (=100%). Mean values and standard error of the means for triplicate measurements from three independent experiments (N=9) are shown. Ctrl=Control, Lz=Letrozole. [file 251517.f1.pdf]
